# Supplementary material for: A fragment-based drug discovery developed on ciclopirox for inhibition of Hepatitis B virus core protein: An in silico study
Source: PLoS One. 2023 May 17;18(5):e0285941. doi: 10.1371/journal.pone.0285941 (PMC10191303; doi:10.1371/journal.pone.0285941)
Supplement: S1 Table — (PDF) [file pone.0285941.s002.pdf]

S1 Table. The atom-atom interactions of Lig118 with the HBV capsid residues

Ligand	Ligand atoms	Interactive residue	Residue number	Residue atoms	Distance (Å)
LIG118	H22	PHE	24	HA	0.52
LIG118	H20	PHE	23	O	0.974
LIG118	H12	TYR	118	OH	0.515
LIG118	H19	PHE	122	CE1	0.468
LIG118	H	PRO	25	HD3	0.448
LIG118	H19	PHE	23	HB3	0.424
LIG118	H23	TRP	102	CE2	0.365
LIG118	C9	LEU	19	HD21	0.349
LIG118	H18	TYR	118	OH	0.338
LIG118	H19	PHE	122	CZ	0.33
LIG118	H14	LEU	19	HD21	0.257
LIG118	H16	LEU	19	HD21	0.241
LIG118	H14	TYR	118	HE2	0.238
LIG118	H3	PRO	25	HG3	0.233
LIG118	H15	LEU	19	HD21	0.232
LIG118	N12	PHE	23	O	0.225
LIG118	H23	TRP	102	NE1	0.215
LIG118	H22	PHE	23	O	0.212
LIG118	C11	PHE	23	HB3	0.159
LIG118	H17	PHE	23	HB2	0.146
LIG118	H21	TYR	118	OH	0.136
LIG118	H17	PHE	23	CB	0.099
LIG118	H	PRO	25	HG3	0.098
LIG118	H21	TYR	118	HE2	0.093

LIG118	C11	PHE	122	CE1	0.092
LIG118	H15	PHE	24	CE1	0.081
LIG118	H	PRO	25	CD	0.065
LIG118	H17	PHE	23	C	0.059
LIG118	H17	PHE	23	O	0.049
LIG118	H20	PHE	23	C	0.047
LIG118	C10	LEU	19	HD21	0.044
LIG118	H20	PHE	23	HB3	0.04
LIG118	H17	PHE	23	HB3	0.029
LIG118	C3	PRO	25	HG3	-0.017
LIG118	H10	TRP	102	NE1	-0.044
LIG118	H10	TRP	102	HE1	-0.048
LIG118	C8	TYR	118	OH	-0.075
LIG118	H15	PHE	24	CD1	-0.082
LIG118	H22	PHE	24	CA	-0.086
LIG118	H19	PHE	23	CB	-0.09
LIG118	H19	PHE	122	HE1	-0.09
LIG118	H1	PHE	23	O	-0.093
LIG118	H23	TRP	102	CD2	-0.133
LIG118	H16	LEU	19	HB3	-0.138
LIG118	C11	PHE	122	CZ	-0.144
LIG118	H	PRO	25	CG	-0.146
LIG118	C14	PHE	24	HA	-0.156
LIG118	C14	PHE	23	O	-0.158
LIG118	C10	PHE	23	HB3	-0.159
LIG118	H22	PRO	25	HD3	-0.16

LIG118	H23	TRP	102	CD1	-0.163
LIG118	H23	TRP	102	CZ2	-0.169
LIG118	C2	PRO	25	HD3	-0.176
LIG118	H15	PHE	24	CZ	-0.184
LIG118	H14	LEU	19	CD2	-0.201
LIG118	C11	PHE	122	CD1	-0.209
LIG118	H19	PHE	122	CD1	-0.214
LIG118	H18	PHE	122	CD1	-0.216
LIG118	C2	PRO	25	HG3	-0.216
LIG118	C11	TYR	118	OH	-0.219
LIG118	H5	PRO	25	HG3	-0.224
LIG118	H12	TYR	118	HH	-0.23
LIG118	C2	PHE	23	O	-0.233
LIG118	H21	TYR	118	CE2	-0.234
LIG118	C10	PHE	23	O	-0.25
LIG118	N12	PHE	23	HB3	-0.258
LIG118	C14	TRP	102	CE2	-0.271
LIG118	C13	PHE	23	O	-0.271
LIG118	H	PHE	23	O	-0.273
LIG118	C11	PHE	23	O	-0.274
LIG118	C9	LEU	19	CD2	-0.274
LIG118	H18	PHE	122	CE1	-0.275
LIG118	C10	PHE	23	HB2	-0.282
LIG118	H16	LEU	19	CD2	-0.294
LIG118	C10	PHE	23	CB	-0.306
LIG118	H16	PHE	122	CD1	-0.307

LIG118	H18	TYR	118	CZ	-0.318
LIG118	H19	PHE	122	HZ	-0.32
LIG118	H22	PHE	23	C	-0.328
LIG118	C9	TYR	118	HE2	-0.335
LIG118	C11	PHE	23	CB	-0.339
LIG118	H14	TRP	102	CZ2	-0.348
LIG118	H21	TYR	118	CZ	-0.349
LIG118	H15	LEU	19	CD2	-0.351
LIG118	H14	TYR	118	CE2	-0.368
LIG118	H13	PHE	122	CZ	-0.369
LIG118	C13	TYR	118	OH	-0.373
LIG118	H18	PHE	122	CG	-0.38
LIG118	H10	TRP	102	CD1	-0.385
LIG118	H5	PRO	25	CG	-0.394
LIG118	C4	PRO	25	HG3	-0.395
